# An evidence-based meta-analysis on the use of brivaracetam in treating seizures in real-world clinical practice

**DOI:** 10.3389/fphar.2025.1716128

**Published:** 2026-01-21

**Authors:** Yu Zhang, Huihui Liu, Xin Wang, Zhiqiang Zhao, Hong Su, Yanjun Ma, Jie Zhao, Qingshan Wang, Liyan Hou

**Affiliations:** 1 Center of Genome and Personalized Medicine, Institute of Cancer Stem Cell, Dalian Medical University, Dalian, China; 2 National-Local Joint Engineering Research Center for Drug-Research and Development (R & D) of Neurodegenerative Diseases, Dalian Medical University, Dalian, China; 3 Jilin Provincial Neuropsychiatric Hospital, Siping, China; 4 First Affiliated Hospital of Dalian Medical University, Dalian, China; 5 Dalian Medical University Library, Dalian, China; 6 College of Health-Preservation and Wellness, Dalian Medical University, Dalian, China; 7 School of Cybersecurity, Liaoning Police College, Dalian, China

**Keywords:** brivaracetam, meta-analysis, real-world study, seizures, systemic review

## Abstract

**Objective:**

The study aimed to evaluate the clinical efficacy and safety of brivaracetam (BRV) as monotherapy or adjuvant therapy for adults with seizures.

**Methods:**

Observational studies of BRV were systematically searched. We estimated the pooled incidence of interests (responder rate, seizure-free rate, adverse effects (AEs), and withdrawal rate) with their corresponding 95% confidence intervals (CIs).

**Results:**

Thirty-five studies, including 10,956 patients, were included. The 50% responder rates were 36% (95% CI: 0.26–0.47), 35% (95% CI: 0.21–0.49), and 40% (95% CI: 0.24–0.56), respectively, and the pooled seizure-free rates were 19.0% (95% CI: 0.17–0.25), 21.0% (95% CI: 0.15–0.27), and 23.3% (95% CI: 0.14–0.31), respectively, at 3, 6, and 12 months of BRV treatment. Subgroup analysis showed that although previous levetiracetam (LEV) failure can affect the response to BRV, treatment with BRV could still be beneficial for these patients. Furthermore, a remarkable reduction in seizure frequency and lower AEs were found among patients who switched directly from LEV to BRV. In addition, BRV, used as the first add-on or monotherapy, displayed a high 50% responder rate and seizure-free rate. The pooled incidence of AEs at 3, 6, and 12 months of BRV treatment were 39.0% (95% CI: 0.27–0.50), 29.0% (95% CI: 0.18–0.39), and 34.0% (95% CI: 0.25–0.44), respectively; the withdrawal rates due to AEs were 7.0% (95% CI: 0.03–0.10), 11.1% (95% CI: 0.05–0.17), and 10.0% (95% CI: 0.07–0.13) at the same follow-up point.

**Conclusion:**

Our meta-analysis has shown BRV to be effective and well-tolerated in both short-term and long-term usage when used as monotherapy or adjuvant therapy for adults with seizures in real-world settings. BRV may be a valuable treatment when used as monotherapy, as a first add-on, and in patients who switched directly from LEV or previously failed to respond to or tolerate LEV.

**Clinical Trial Registration:**

https://www.crd.york.ac.uk/PROSPERO/, identifier CRD420251085564.

## 1 Introduction

Epilepsy is one of the most common and chronic neurological disorders, affecting approximately 70 million people worldwide and resulting in significant risks to individual health and reduced quality of life (Beghi et al., 2019). Currently, anti-seizure medications (ASMs) are the first-line therapy. ASM monotherapy is the recommended initial therapeutic strategy for patients who are newly diagnosed with epilepsy while reducing adverse events (AEs). However, approximately 40%–50% of individuals with epilepsy fail to achieve seizure freedom with their initial monotherapy (Brodie et al., 2012). Many of these people have to use alternative monotherapy or combination therapies with one or more additional ASMs (Xiao et al., 2018). Despite the number of new approved ASMs increasing dramatically over the past 20 years, more than 30% of patients cannot obtain optimal efficacy or are unable to tolerate pharmacotherapy due to severe AEs (Lattanzi et al., 2022b). Therefore, it is urgent to develop novel and more effective ASMs to provide new therapeutic options for patients with refractory epilepsy.

Brivaracetam (BRV) is a third-generation ASM that was approved as an adjunctive treatment for focal seizures in adults and adolescents over 16 years old in many countries, such as the European Union and the United States (Brandt et al., 2016; Lattanzi et al., 2022c). BRV is a high-affinity ligand of the synaptic vesicle protein 2A (SV2A), similar to levetiracetam (LEV), but it shows 15- to 30-fold higher binding affinity, with a faster brain penetration and onset of action (Gillard et al., 2011; Klitgaard et al., 2016). Previous meta-analysis synthesizing data from phase-II and phase-III randomized, double-blind trails demonstrated that adjunctive BRV 5 mg–200 mg/day might be efficacious and well-tolerated in adults with refractory partial-onset seizures compared with placebo (Lattanzi et al., 2016). However, these RCTs have some limitations. For example, little is known regarding whether the outcome of BRV is effective in patients with intellectual disability (ID) and those with structural brain and post-stroke epilepsy since they are often excluded from RCTs due to legislation; however, those patients are an important group in routine clinical practice. In addition, results from RCTs do not provide sufficient information to extrapolate to everyday clinical practice (Brandt et al., 2020). Additional post-marketing data on the effectiveness of BRV when it is used as a monotherapy or first add-on therapy in real-world settings is needed urgently. To date, numerous real-world studies about BRV have been reported; however, findings regarding its seizure-control efficacy have resulted in some inconsistency due to small sample sizes and variations in study designs. For instance, one study showed that up to 60% of patients who were switched from LEV re-switched to LEV because BRV was not more efficacious than LEV (Steinhoff et al., 2017). However, some studies indicated that BRV might be more efficacious and better tolerated in the treatment of epilepsy than LEV. Since data from post-marketing observational studies can provide further important insight into how to best utilize the BRV under real-life conditions, a systematic review and meta-analysis are required to better understand the efficacy and safety of BRV in patients with epilepsy.

Therefore, this systematic review and meta-analysis aimed to synthesize the existing evidence from post-marketing observational studies to evaluate the clinical efficacy and safety of BRV as monotherapy or adjuvant therapy for adults with various epilepsy syndromes. We hope that the results of this study will provide further accurate and updated evidence of the role of BRV for its clinical application for adult epilepsy patients.

## 2 Methods

### 2.1 Literature search strategy

Our meta-analysis was conducted according to the Preferred Reporting Items for Systematic Reviews and Meta-Analyses (PRISMA) principles (Fisher et al., 2017). This study was prospectively registered in PROSPERO under the registration number CRD420251085564 in https://www.crd.york.ac.uk/PROSPERO/. To find potentially eligible articles, the PubMed, EMBASE, and Cochrane Central Register of Controlled Trials (CENTRAL) databases were systematically searched from inception to June 2024. Subsequent searches were run on 1 May 2025. The search terms were selected by combining the MeSH words “brivaracetam” and “epilepsy” with corresponding free words in the title/abstract. The details of the literature retrieval strategy for the electronic databases are outlined in Supplementary Table S1. Additionally, we manually searched the reference lists from all included eligible articles for other potentially relevant studies.

### 2.2 Inclusion and exclusion criteria

Articles were included in this meta-analysis if they met the following criteria: 1) adult participants (aged 16 years or older) diagnosed with epilepsy; 2) real-world studies (excluding RCTs and *post hoc* analyses) with a treatment period (excluding titration of a minimum of 4 weeks) of at least 8 weeks; 3) original studies reporting the primary outcome data of at least one of the following: 50% responder rate, seizure freedom rates, withdrawal rate due to adverse events (AES), or AE rate; 4) more than 10 patients were present in each arm at the end of the follow-up period to reduce bias; and 5) BRV was given as monotherapy or adjunctive treatment combined with other ASMs.

The exclusion criteria were as follows: 1) literature such as reviews, letters, case reports, or conference abstracts; 2) studies that were conducted on children, healthy volunteers, or BRV as the treatment for conditions other than epilepsy; 3) preclinical studies (i.e., animal or cell-based studies); and 4) studies that had missing dates or were unable to extract effective outcome indicators.

### 2.3 Outcome measures

The efficacy outcomes of this meta-analysis were a 50% responder rate (defined as the proportion of patients whose seizure frequency was 50%–99% lower than that in the baseline period), seizure-free rate (defined as the proportion of patients who had no seizure relative to baseline), and retention rates (defined as the proportion of patients who continued BRV treatment until the follow-up period) after BRV treatment.

Safety and tolerability outcomes were the AE rate (defined as the proportion of patients who experienced at least one AE regardless of relevance with ASMs), the withdrawal rate due to AEs (defined as the proportion of patients with AEs leading to discontinuation), and the incidence of AEs reported by ≥2 studies. Notably, the intent-to-treat (mITT) population set (in the mITT analysis, all subjects who took ≥1 dose of the study medication and had documented seizure frequency data throughout the BRV treatment duration were included) was used in this meta-analysis.

Given that the efficacy and safety of BRV could be affected by several factors, subgroup analyses were performed. Comparisons between subgroups based on previous LEV usage, study duration (short-term follow-up, including 3 months and 6 months, and long-term follow-up ≥12 months), BRV add-on therapeutic schedule (early vs. late add-on therapy), and the use of ID were performed. In addition, the use of BRV as monotherapy and switching directly from LEV to BRV were assessed in this study.

### 2.4 Study selection, data extraction, assessment of the risk of bias, and confidence in the evidence

Two reviewers (Huihui L and Xin W) independently screened all the titles and abstracts of all the articles, marking them as “exclude” or “include.” Discrepancies of a selected article were discussed with the senior authors (Qingshan W) until a consensus was reached. Subsequently, two reviewers (Huihui L and Xin W) independently assessed the full text of each article according to the inclusion and exclusion criteria specified above. The extracted data included the title, first author and date of publication, patients’ demographic information, outcome measures (efficacy and safety outcomes described above), and trial design (duration of follow-up, concomitant ASMs, and dosage of BRV). The risk of bias was assessed using the Newcastle–Ottawa Scale (NOS) to evaluate the quality of all potentially eligible studies, with a maximum score of nine points. The studies with a score of ≥7 points were recognized as high quality, and those with ≤5 points were considered low-quality and, therefore, excluded (Stang, 2010).

### 2.5 Statistical analysis

Statistical analysis was performed using STATA version 17.0 (StataCorp LP, College Station, TX). We estimated the pooled incidence of endpoints of interest with their corresponding 95% confidence intervals (CIs). I^2^ and Cochran’s Q test were used to evaluate the degree of between-study heterogeneity. When *p* > 0.1 and I^2^ < 40%, a fixed-effects model was used to calculate the pooled rates (PRs) with 95% CI; otherwise, a random-effects model was used (I^2^ ≥ 40% or *p* ≤ 0.1). The potential publication bias was analyzed using Egger’s test, and *p* < 0.05 indicated that there was significant publication bias.

## 3 Results

### 3.1 Results of the search and the characteristics of the included studies

**FIGURE 1:**
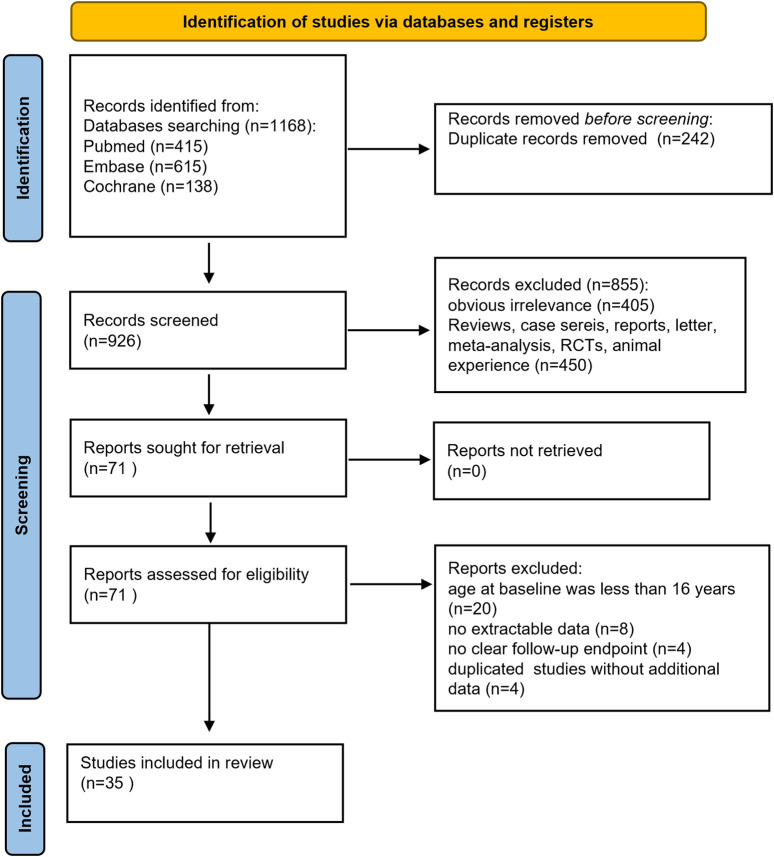
Flow diagram of the study process.

A total of 1,168 documents were obtained through electronic retrieval. Duplicate articles were deleted using EndNote 9.0 software, and 926 articles were retained after the removal of duplicates. Further screening of the titles and abstracts of the literatures excluded 405 articles that were irrelevant. Another 450 articles were excluded because they were reviews, commentaries, conference abstracts, animal- or cell-based studies, or RCTs and *post hoc* analyses of RCTs, leaving 71 studies for further review. After full-text assessment, 36 articles were excluded for several reasons, including 20 articles involving children, 8 articles with no extractable data, 4 studies with no clear follow-up endpoint, and 4 studies with duplicated results providing no additional data. Finally, 35 studies were included in this meta-analysis (Abraira et al., 2021; Adewusi et al., 2020; Andres et al., 2018; Arnold et al., 2020; Depondt et al., 2021; Faught et al., 2024; Fonseca et al., 2023; Fonseca et al., 2020; Gillis et al., 2021; Green et al., 2022; Halliday et al., 2023; Hirsch et al., 2018; Lafortune et al., 2020; Lattanzi et al., 2022; Lattanzi et al., 2023; Lattanzi et al., 2022; Lattanzi et al., 2021; Lattanzi, 2022a; Lattanzi et al., 2024; Lerche et al., 2020; Liguori et al., 2020; Roberti et al., 2024; Savastano et al., 2021; Siddiqui et al., 2023; Snoeren et al., 2022; Steinhoff et al., 2017; Steinhoff et al., 2020; Steinig et al., 2017; Stephen et al., 2021; Svendsen et al., 2022; Toledo et al., 2021; Villanueva et al., 2019; Villanueva et al., 2024; Yanes et al., 2023; Yates et al., 2015). The selection process of the included studies is presented in Figure 1. The characteristics of the patients are shown in Table 1. Overall, the included studies evaluated the effectiveness and safety of BRV in adults with various epilepsy syndromes. All studies were published between 2018 and 2024. A total of 10,095 patients were enrolled in this meta-analysis. A total of 33 studies compared the effect of BRV as add-on therapy with baseline, while three studies assessed BRV as add-on and monotherapy treatment for epilepsy. Except for two studies where the subjects were much older than in the other studies, patients across the studies were similar in terms of age, sex ratio, and concomitant ASMs. Since only one study examined the long-term efficacy of BRV (follow-up ≥12 months), we analyzed BRV treatment effects at 3, 6, and 12 months in comparison to baseline.

**TABLE 1:** Characteristics of the observational studies included in this meta-analysis.

First author and year	Number of patients	Study design	Type of concomitant AED	Gender (male/female)	Age, years [mean ± SD or mean (range)]	Duration of epilepsy (years/months ± SD)	Duration of follow-up (months)	BRV dosage (mg/day) [mean ± SD or mean (range)]	Seizure classification, number of patients			
									A	B	C	D
Abraira et al. (2021)	41	Add-on	OXC, LTG, and clobazam	21/20	40.9 ± 17.8	17.0 ± 12.4	6	142.0 ± 47.0	SS, 25	GG, 4	UC, 10	DEE, 2
Adewusi et al. (2020)	290	Add-on	NA	134/156	38 (15–77)	21 (2–56)	3, 6, ≥12	150 (20–500)	LRE, 193	GS, 47	UC, 50	​
Andres, et al. (2018)	33	Add-on	LCM, OXC, VPA, and ZNS	19/14	38.2 (17–63)	NA	6, 12	50–200	LRE, 32	GS, 1	​	​
Arnold, et al. (2020)	108	Add-on and monotherapy	LEV, CBZ, LTG, OXC, LCM, and ZNS	52/56	40.8 ± 13.0	18.8 ± 14.0	3, 6, 12	100–200	LRE, 108	CPS, 96	FBTCS, 66	GS, 7
Depondt, C.(2021)	175	Add-on	LEV, LCM, and CBZ	79/96	38.0 ± 13.4	NA	3, 6, 9	NA	DRE, 175	​	​	​
.Fonseca et al. (2023)	123	Add-on	LCM, eslicarbazepine acetate, LTG, clonazepam, and valproate	64/59	36 3 ± 18 0.0	13 0 ± 11 8	3, 6, 12	75 (25–300)	DRE, 63	LRE, 78	IGE, 45	​
Fonseca et al. (2020)	37	Add-on and monotherapy	VPA, LTG, and clobazam	10/27	29.9 ± 12.3	11.1 (1.2–40.9)	6, ≥12	135.1 ± 79.2	GTCS, 22	Absences, 11	MS, 4	​
Gillis et al. (2021)	116	Add-on	NA	65/51	34.9 ± 14.5	NA	3, 6, 12	88.1 (20–200)	LRE, 77	GS, 13	sGS, 26	​
Green et al. (2022)	200	Add-on	OXC and LTG valproate and perampanel ZNS and LCM OXC	80/120	41.0 ± 14	14.1 ± 12	6, 12, 18, 36	100–200	FIAS, 143	Absence, 26	TC, 33	​
Hirsch et al. (2018)	61	Add-on	NA	NA	NA	NA	6	153.2 ± 74.8	NA	​	​	​
Lafortune et al. (2020)	38	Add-on	NA	10/28	36.2 ± 13	22.6 ± 13	3, 6, 12	88.1 (20–200)	LRE, 32	GS, 6	​	​
Lattanzi et al. (2022)	176	Early add-on	NA	85/91	45 (30–61)	11 (5–25)	12	125 (100–200)	LRE, 114	FBTCS, 32	LRE-FBTCS, 11	​
	853	Late add-on	NA	402/451	45 (33–55)	27 (16–39)	12	200 (100–200)	LRE, 565	FBTCS, 139	LRE-FBTCS, 55	​
Lattanzi et al. (2022)	75	Add-on	NA	36/39	57 (42–66)	10 (4–21)	3, 6, 12	100 (100–200)	LRE, 48	FBTCS, 15	LRE-FBTCS, 5	​
Lattanzi et al. (2023)	506	Add-on	NA	NA	NA	NA	12	150 (100–200)	NA	​	​	​
Lattanzi et al. (2022)	918	<65	NA	436/482	42 (31–52)	25 (14–37)	12	150 (100–200)	LRE, 599	FBTCS, 156	LRE-FBTCS, 61	​
	111	≥65	NA	51/60	69 (67–74)	23 (8–51)	12	150 (100–200)	LRE, 80	FBTCS, 15	LRE-FBTCS, 5	​
Lattanzi et al. (2021)	1029	Add-on	CBZ and VPA LCM	487/542	45 (33–56)	25 (13–38)	3, 6, 12	150 (100–200)	LRE, 679	FBTCS, 171	LRE-FBTCS, 66	​
Lattanzi et al. (2024)	44	Monotherapy	NA	18/26	63.5 (44–73.5)	7.5 (1.5–18.5)	6, 12	150 (100–200)	LRE, 26	FBTCS, 13	LRE-FBTCS, 5	​
Lerche et al. (2020)	506	Add-on	LEV and LCM VPA and ZNS TPM and OXC LTG	265/241	41.6 ± 15.9	24.9 ± 16.1	6	200 (50–400)	LRE, 390	SGS, 384	​	​
Liguori et al. (2020)	43	Add-on	VPA and OXC LTG and TPM CBZ and LCM	21/22	42.32 ± 15.78	18.12 ± 14.86	12	116.5 ± 38.23	LRE, 25	GG, 9	sGS, 9	​
Roberti et al. (2024)	165	LEV-	LEV and VPA CBZ	78/87	43 (30–53.5)	70 (149.3–429)	6, 12, 24, 36	150 (100–200)	LRE, 114	GS, 50	sGS, 221	​
	77	LEV+	LEV and VPA CBZ	36/41	7 (33.5–54)	24 (132.5–474)	6, 12, 24, 36	200 (150–200)	LRE, 59	GS, 6	sGS, 44	​
Savastano et al. (2021)	76	Add-on	CBZ and VPA LTG and TPM OXC and LCM	35/41	42 ± 15	23.15 ± 13.2	6	25–200	FIAS, 20	FWAS, 10	Focal to bilateral, 46	​
Siddiqui et al. (2023)	368	Add-on	NA	287/181	32.91 ± 17.11	NA	3	25–100	LRE, 368	​	​	​
Snoeren et al. (2022)	379	Add-on	NA	184/195	42.3 (18–82)	25.4 (1–75, ±15.2)	3, 6, 12	144.2 (20–400, 66.4)	LRE, 314	GS, 37	sGS, 6	Unknown, 197
Steinhoff et al. (2017)	101	Add-on	NA	47/54	42 (18–81)	NA	3, 6	168.6 (50–400)	GTCS, 3	POS, 98	sGTCS, 86	​
Steinhoff et al. (2020)	261	Add-on	LTG and LEV LCM and CBZ ZNS	130/131	42.0 ± 13.2	23.2 ± 14.1	3, 6	100 (0–400)	LRE, 229	​	​	​
Steinig et al. (2017)	262	Add-on	NA	129/133	40.0 ± 16.0	21.6 ± 14.7	3, 6, 12	100 (25–400)	LRE, 227	IGE, 19	SGE, 8	UC, 8
Stephen et al. (2021)	108	Add-on and monotherapy	CBZ and VPA LCM and LTG,	38/70	45 (17–78)	NA	6	100 (25–200)	LRE, 88	GGEs, 20	GTCS, 2	GTCS/absence, 2
Toledo et al. (2021)	753	Add-on	CBZ and LTG LCM and OXC TPM and VPA	357/396	17.5 ± 13.6	22.7 ± 13.9	6, 12	200	SPS, 289	CPS, 584	FBTCS, 184	​
Villanueva et al. (2019)	575	Add-on	LEV and CBZ LTG .	290/285	41.9 (16–88)	14 (5.0–27.0)	3, 6, 12	187.5, (25–350)	SPS, 132	CPS, 391	SGS, 181	​
Yanes et al. (2023)	51	Add-on	LCM and CBZ VPA and OXC LEV	26/25	37 (30–52)	NA	6	100	SPS, 22	CPS, 36	FBTCS, 17	​
Yates et al. (2015)	29	Add-on	LTG and TPM CBZ and LCM OXC.	15/14	35.8 ± 11.8	16.2 ± 12.7	3	200 (50–200)	POS, 22	GS, 9	UC, 1	​
Halliday et al. (2023)	228	Add-on	LCM and LTG VPA	102/126	41 (30–50)	NA	3, 6, 12	100 (25–200)	LRE, 187	IGE, 25	DEE, 11	Unknown, 5
Svendsen et al. (2022)	120	Add-on	LTG	49/71	37 (17–91)	24 (2–61)	12	150 (25–200)	LRE, 89	GS, 25	UC, 6	​
Villanueva et al. (2024)	276	Monotherapy	NA	119/157	53.9 (18–92)	46 (0–90)	3, 6, 12	25–400	Structual, 139	Other, 70	Genetic, 20	Unknown, 114
Faught et al. (2024)	1644	Add-on	LEV	790/853	≥16	≥65 years: 33 (10–58.6) 16–65 years: 17 (8–28)	3, 6, 12	100 (50–100)	POS, 1515	POS with secondary, 675	Genetic, 127	Unknown, 13

GGEs, genetic generalized epilepsies; FWAS, focal with awareness seizure; GG, generalized genetic; SS, structural seizure; AEDs, anti-epileptic drugs; SD, standard deviation; TC, Tonic–clonic; NA: not available; SPS, simple partial seizure; CPS, complex partial seizures; FBTCS, focal to bilateral tonic–clonic seizures; POS, partial-onset seizures; sGTCS, secondarily generalized tonic–clonic seizures; UC, unclassified; GTCS, generalized tonic–colic seizures; MS, myoclonic seizure; SGS, secondarily generalized seizures; DRE, drug-resistant epilepsy; CLD, patients with cognitive/learning disability; IGE, idiopathic generalized epilepsy; SGE, symptomatic generalized epilepsy; GS, generalized seizures; DEE, epileptic encephalopathy; FIAS, focal impaired awareness seizure; sGS, focal and generalized seizures; LRE, localization-related epilepsy (focal epilepsy).

Abbreviation of concomitant drugs: LEV, levetiracetam; TPM, topiramate; VPA, valproic acid; CBZ, carbamazepine; OXC, oxcarbazepine; LCM, lacosamide; LTG, lamotrigine; ZNS, zonisamide.

### 3.2 Clinical efficacy outcomes

**FIGURE 2:**
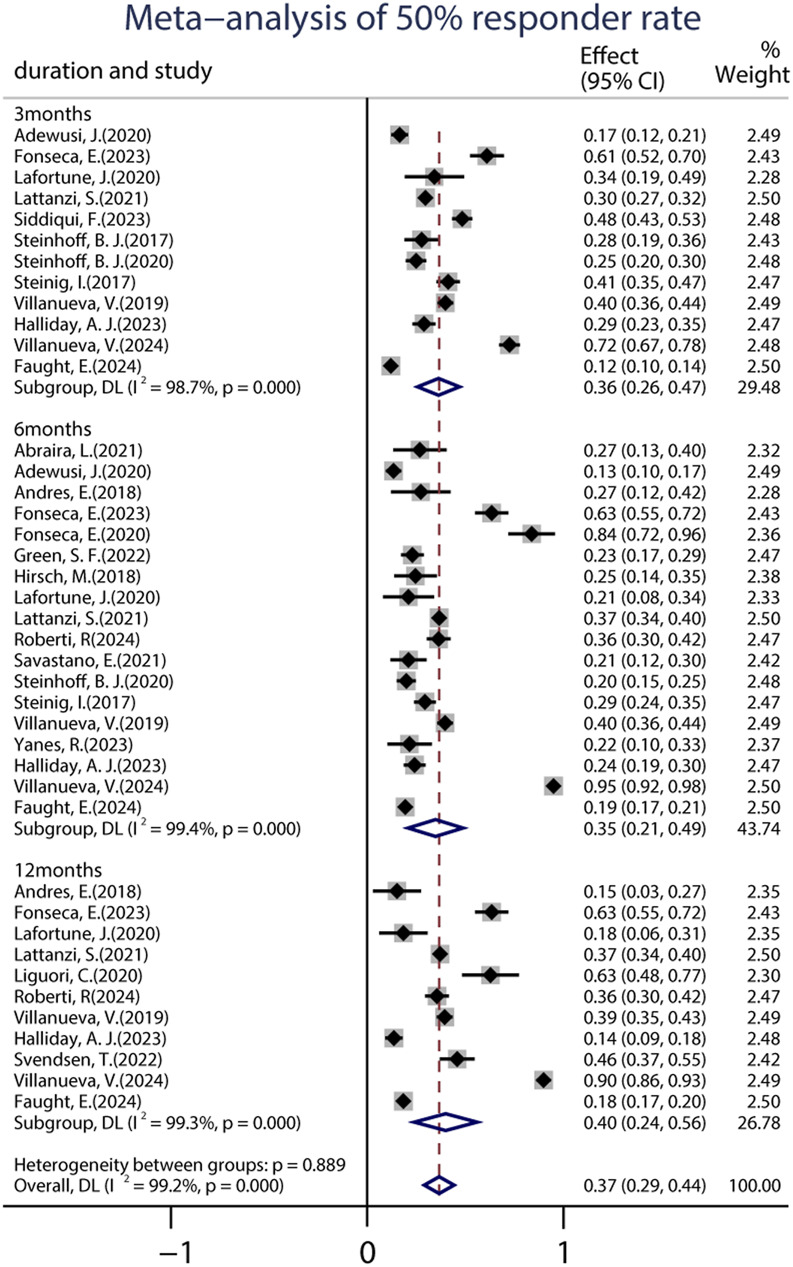
Meta-analysis of the 50% responder rate.

**FIGURE 3:**
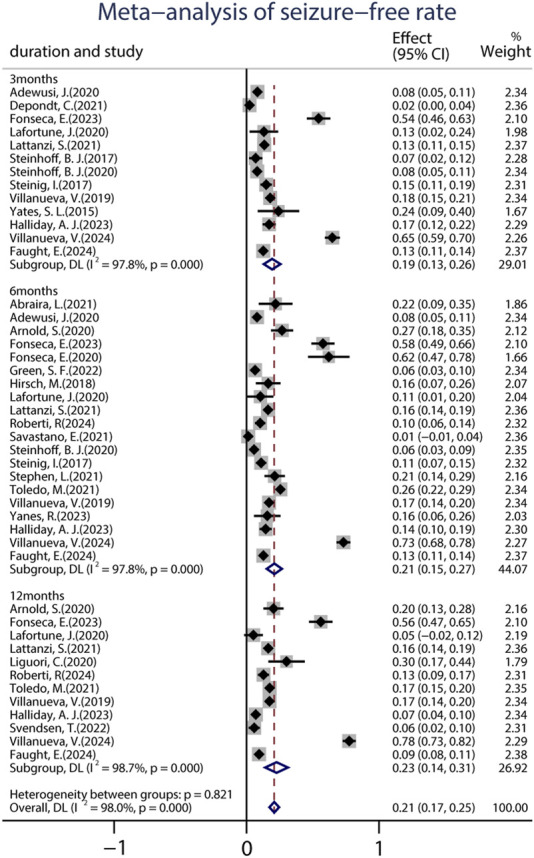
Meta-analysis of the seizure-free rate.

A total of 22 studies provided data on the 50% responder rates, and the original responder rates of BRV treatment, ranging from 13.60% to 83.78% were available for analysis for 5,979 patients. Because a significant heterogeneity (heterogeneity: *p* = 0.889, I^2^ = 99.2%) was observed between the included studies, a random-effects model was used to calculate the pooled relative risk (RR) and corresponding 95% CI. The pooled 50% responder rate was 0.37 (95% CI: 0.29, 0.44) (Figure 2) in all the studies. Because 12 studies were carried out at different time-points, we analyzed the data separately according to the length of follow-up (3, 6, and 12 months after BRV treatment) and treated them as separate datasets. The pooled responder rates were 0.36 (95% CI: 0.26–0.47) and 0.35 (95% CI: 0.21–0.49) after 3 and 6 months of BRV treatment, respectively (Figure 2). Notably, 11 studies provided responder rates in response to long-term (12-month follow-up) BRV treatment. Further analyses of long-term BRV treatment showed a pooled RR of 0.40 (95% CI: 0.24–0.56). No publication bias was observed in the meta-analyses based on Begg’s test (*p* = 0.20). A total of 25 studies reported the seizure-free rate in the follow-up period compared with the baseline, including 6,884 patients during BRV treatment. The pooled seizure-free rate was 21.0% (95% CI: 0.17–0.25) (Figure 3) in all studies; the pooled seizure-free rate was 19.0% (95% CI: 0.13–0.26) and 21.0% (95% CI: 0.15–0.27) after 3 and 6 months of BRV treatment with high heterogeneity (Figure 3). Furthermore, 12 studies reported on the seizure-free rate in response to long-term perampanel (PER) treatment. The pooled seizure-free rate at 12 months of follow-up was 23.3% (95% CI: 0.14–0.31) with high heterogeneity (Figure 3).

A total of 27 studies reported data on the proportion of patients who continued treatment at the end of the follow-up period, including 8,032 patients. The pooled retention rate from the random-effects model was 85.0% (95% CI: 0.80–0.90), 73.0% (95% CI: 0.68–0.78), and 65.0% (95% CI: 0.59–0.71) at 3, 6, and 12 months of PER treatment, respectively (Supplementary Figure S1).

#### 3.2.1 Impact of early or late add-on on the efficacy of BRV

Two studies (including 1,072 patients) compared the effects of BRV as an add-on therapy when administered after 1–2 (early add-on) and ≥3 (late add-on) prior to other anti-seizure medications. The pooled 50% responder rate was 64.7% (95% CI: 0.43–0.86) in the early add-on group and 36.5% (95% CI: 0.25–0.48) in the late add-on group, respectively. Similar to the 50% responder rate, patients in the early add-on group (30.8%, 95% CI: 024–0.39) displayed a higher seizure-free rate than those in the late add-on group (10.8%, 95% CI: 0.09–0.13) after the 12-month study period.

#### 3.2.2 Efficacy of BRV in patients with learning disability

**FIGURE 4:**
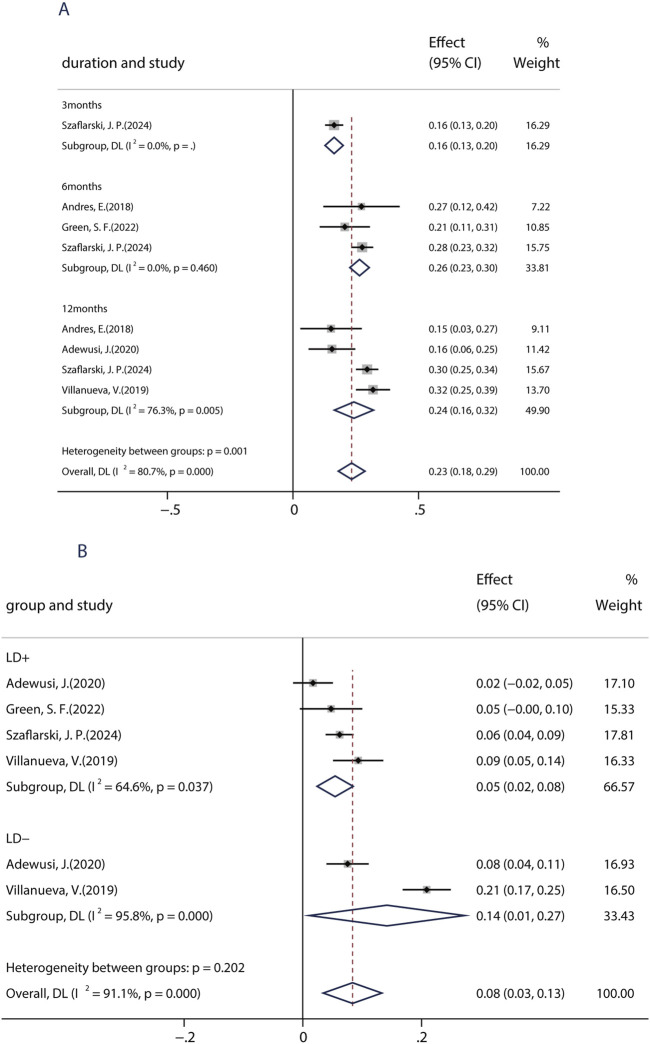
Efficacy of BRV in patients with learning disability. **(A)** 50% responder rate of BRV in patients with learning disability. **(B)** Seizure-free rate of BRV in patients with learning disability.

Patients with learning disabilities (LDs) are often excluded from RCT studies, so there is a lack of information on the efficacy and tolerability of BRV in such patients. Until now, a total of five studies (including 739 patients) assessed the effects of BRV in patients with epilepsy and LD. Among those studies, two studies compared the efficacy of BRV in patients with LD (LD+ group) and patients without LD (LD− group), whereas the remaining three studies evaluated its effects in patients with LDs. The pooled 50% responder rate in patients with IDs was 23.4% (95% CI: 0.18, 0.29) across all studies. Subgroup analysis showed that the pooled responder rates were 16.4% (95% CI: 0.13–0.20) and 26.5% (95% CI: 0.23–0.31) after 3 and 6 months of BRV treatment, respectively (Figure 4A). Notably, the pooled responder rate was 24.3% (95% CI: 0.17–0.32) from four studies for long-term (12-month follow-up) treatment. Further analysis indicated that the outcome in patients without LD was somewhat better than that in those with LD: the pooled ≥50% responder rate was 29% in the LD− group and 24% in the LD+ group. Then, the pooled RR with its corresponding 95% CI for the LD+ and LD− groups was calculated. No statistical difference was observed (RR = 0.83, 95% CI: 0.65–1.06), suggesting that patients with or without LD display similar 50% responder rates after BRV treatment. The pooled seizure-free rate was 14.2% (95% CI: 0.01–0.27) and 0.54% (95% CI: −0.02–0.85) for the patients in the LD− and LD+ groups, respectively (Figure 4B). The estimated RR was 0.47 (95% CI: 0.29–0.75) with low heterogeneity (I^2^ = 0.0%, p = 0.80), indicating that patients in the LD− group displayed a higher seizure-free rate than those in the LD+ group.

#### 3.2.3 Impact of previous LEV-exposure on the efficacy of BRV

**FIGURE 5:**
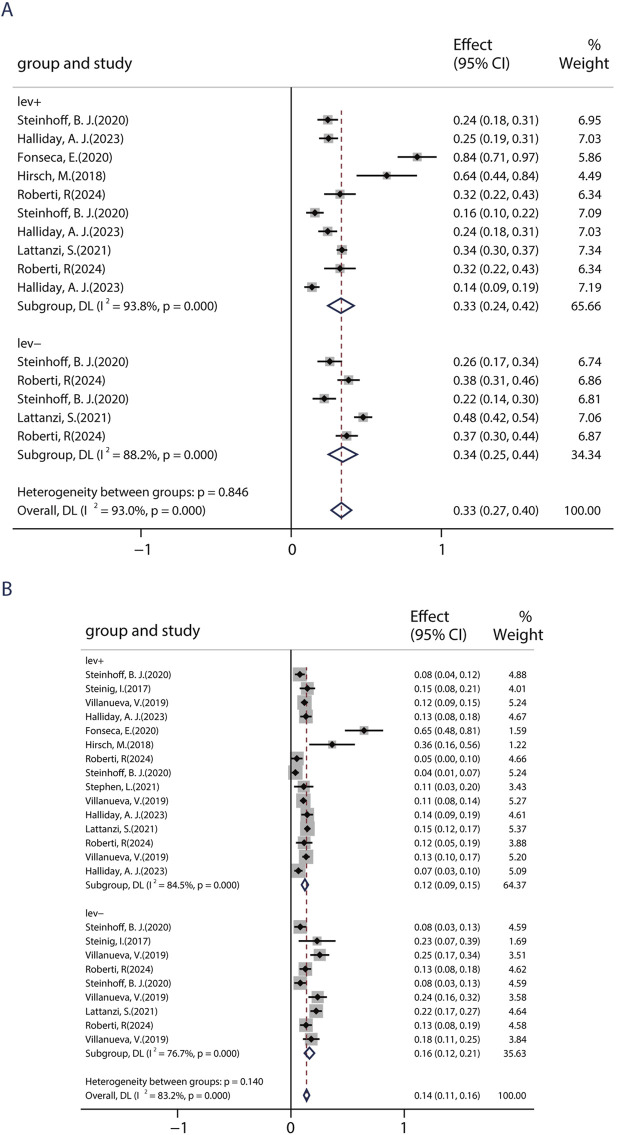
Impact of previous levetiracetam-exposure on BRV efficacy. **(A)** 50% responder rate of BRV in patients if they had previously used LEV. **(B)** Seizure-free rates of BRV in patients if they had previously used LEV.

Evidence demonstrates that combining ASMs with distinct mechanisms of action (MOAs) can attain seizure control, including seizure freedom, in patients who are unresponsive to initial antiepileptic regimens. In this study, patients were placed in the LEV+ group if they had previously used LEV and in the LEV− group if they had not. Six studies, including 1,698 patients, showed a 50% responder rate of BRV in patients with/without previous LEV exposure. Among six studies, five studies compared the efficacy of BRV in patients with prior use of LEV (LEV+ group) and patients without prior use of LEV (LEV− group). The pooled responder rates were 33.1% (95% CI: 0.24–0.42) in patients with prior use of LEV and 34.4% (95% CI: 0.25–0.44) in LEV-naive patients (Figure 5A). Nine studies, including 2,405 patients, showed the seizure-free rate of BRV in patients with/without previous LEV exposure. Among nine studies, five studies compared the efficacy of the BRV in two groups. The pooled seizure-free rates were 16.4% (95% CI: 0.12–0.21) and 12.4% (95% CI: 0.09–0.15) for the patients in the LEV− and LEV+ groups, respectively (Figure 5B). The estimated RR was 0.65 (95% CI: 0.54–0.77) with low heterogeneity (I^2^ = 0.0%, *p* = 0.32), indicating that patients in the LEV− group displayed a higher seizure-free rate than those in the LEV+ group. Similar to the 50% responder rate, patients in the LD− group (14.2%, 95% CI: 0.01–0.27) displayed a higher seizure-free rate than those in the LD+ group (0.54%, 95% CI: −0.02–0.13).

**FIGURE 6:**
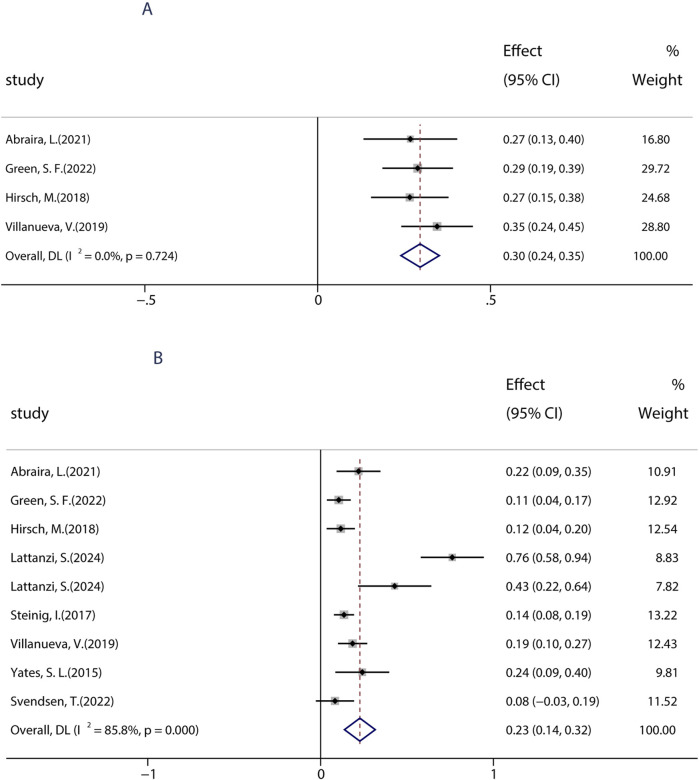
Efficacy of an overnight switch from LEV to BRV. **(A)** 50% responder rate in patients who switched from LEV to BRV overnight. **(B)** Seizure-free rates in patients who switched from LEV to BRV overnight.

Previous studies showed that an immediate switch from LEV to BRV may improve therapeutic effects and decrease the incidence of AEs. Given the similar action of BRV and LEV, further analyses of BRV related to the switch were performed. To date, nine studies including 488 patients assessed the efficacy of an overnight switch from LEV to BRV. The pooled 50% responder rate in patients who immediately (or overnight) switched from LEV to BRV was 30.0% (95% CI: 0.24, 0.35) (Figure 6A) in all studies, and the pooled seizure-free rate was 23.0% (95% CI: 0.14, 0.32), indicating that switching from LEV to BRV is an effective therapeutic option (Figure 6B).

#### 3.2.4 Impact of monotherapy on the efficacy of BRV

**FIGURE 7:**
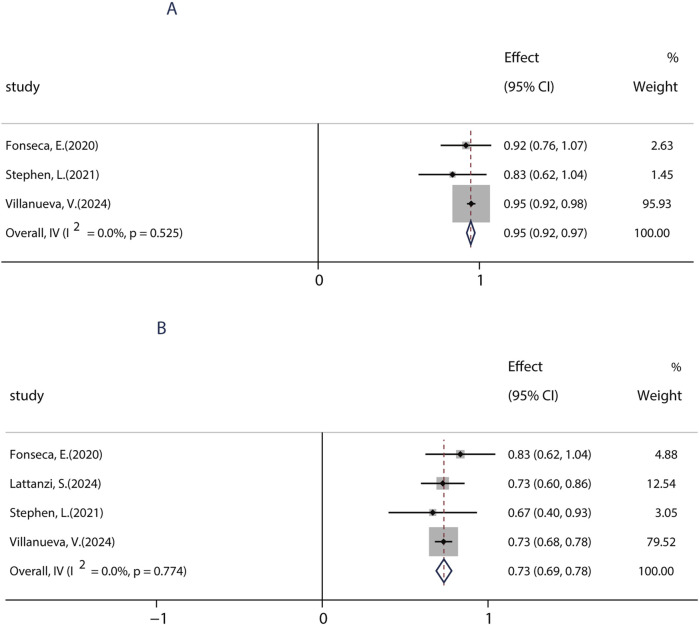
Efficacy of BRV as monotherapy. **(A)** Pooled 50% responder rate of PER as monotherapy. **(B)** Pooled seizure-free rate of PER as monotherapy.

To date, BRV can be prescribed as monotherapy for focal epileptic seizures (FOS) only in countries such as the United States, Japan, and China. To date, only one research evaluated the efficacy of BRV as the primary monotherapy (patients were treated with BRV only in the absence of any other concomitant ASMs) in seizures. A total of four studies performed from 2020 to 2024 were used to evaluate the impact of monotherapy on the efficacy of BRV in patients with FOS, which were conducted in patients receiving PER as the conversion monotherapy. Because most included studies examined the effects of BRV treatment at 6 months compared to baseline, we analyzed the efficacy of BRV monotherapy at that time-point. The combined outcomes showed that the 50% responder rate and the seizure-free rate were 95.0% (95% CI: 0.92–0.97) and 73.3% (95% CI: 0.69–0.78), respectively (Figure 7).

### 3.3 Clinical safety outcomes

**FIGURE 8:**
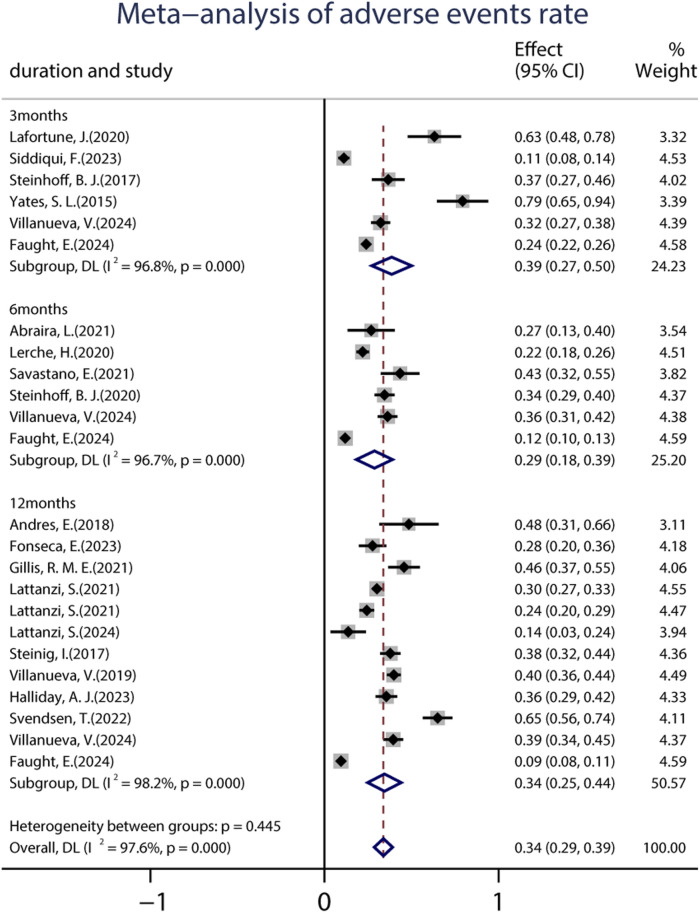
Meta-analysis of the adverse events rate.

**FIGURE 9:**
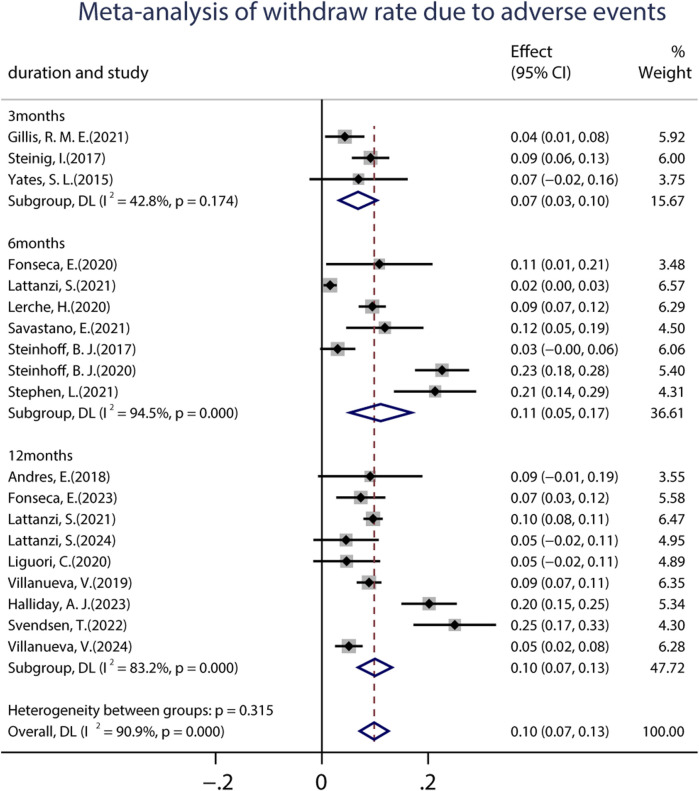
Meta-analysis of the withdrawal rate due to adverse events.

A total of 19 studies (including 6,160 patients) provided data on the proportions of participants who experienced at least one treatment-emergent adverse event (TEAE) after receiving at least one dose of BRV. The pooled incidence of AEs from the random-effects model indicated that it was 34.0% (95% CI: 0.29–0.39; *p* = 0.00) (Figure 8), regardless of the length of follow-up. Subgroup analysis showed that the pooled incidences of TEAEs at 3 and 6 months were 39.0% (95% CI: 0.27–0.50) and 29.0% (95% CI: 0.18–0.39), respectively (Figure 8). Further analyses of the incidence of pooled long-term (12-month follow-up) AEs were 34.0% (95% CI: 0.25–0.44). A total of 18 studies (including 3,967 patients) provided the withdrawal rate owing to AEs. The pooled withdrawal rates at 3, 6, and after 12 months of BRV treatment were 7.0% (95% CI: 0.03–0.10), 11.1% (95% CI: 0.05–0.17), and 10.0% (95% CI: 0.07–0.13), respectively (Figure 9).

We further performed a meta-analysis on 24 AEs (shown in Table 2). In the pooled analysis, the common AEs that were reported in more than nine studies (more than 50% of studies provided data on AEs) were dizziness (6%, 95% CI: 0.04–0.08), somnolence (9%, 95% CI: 0.06–0.11), depression (5%, 95% CI: 0.04–0.07), aggression (4%, 95% CI: 0.03–0.06), fatigue/tiredness (5%, 95% CI: 0.03–0.07), headache (2%, 95% CI: 0.01–0.04), and irritability (5%, 95% CI: 0.03–0.07). It should be noted that other AEs that were reported only in a small number of patients, such as suicidal ideation, sleep disturbance, cognitive decline, ataxia/instability, and psychosis, might also affect the tolerability of BRV. In addition, one study mentioned some rare AEs, including personality change, paranoia, allergy, swelling, edema, blurred vision, and urinary incontinence.

**TABLE 2:** Summary of AEs reported in ≥2 studies in the meta-analysis.

System organ class	Preferred term	No. of studies	Total patients with the AE/total patients treated with BRV	Pooled rates (95% CI)
Nervous system disorders				
​	Dizziness	16	327/5,441	0.06 (0.04–0.08)
	Somnolence	14	337/5,092	0.09 (0.06–0.11)
	Headache	10	80/3,093	0.02 (0.01–0.04)
	Cognitive decline	9	159/4,453	0.04 (0.02–0.06)
	Sleep disturbance	8	48/2,938	0.01 (0.01–0.02), fixed
	Ataxia/instability	6	26/1,301	0.02 (0.01–0.02)
	Vertigo	5	72/2,448	0.03 (0.01–0.04)
	Sedation	3	16/414	0.05 (−0.01–0.11)
	Tremor/parkinsonism	3	8/567	0.01 (0.01–0.02), fixed
Psychiatric disorders				
​	Depression	12	124/2,553	0.05 (0.04–0.07)
	Aggression	11	146/3,447	0.04 (0.03–0.06)
	Irritability	11	197/3,906	0.05 (0.03–0.07)
	Agitation/nervousness	5	91/2,272	0.03 (0.01–0.06)
	Mood change	4	36/1,834	0.02 (0.00–0.03)
	Anxiety	5	67/2,795	0.03 (0.01–0.04)
	Psychosis	2	13/382	0.04 (−0.03–0.11)
	Confused state/mental slowing	2	9/363	0.02 (0.01–0.04), fixed
	Suicidal ideation/attempt	2	4/523	0.01 (−0.00–0.01), fixed
General disorders and administration site conditions				
​	Fatigue/tiredness	13	227/5,085	0.05 (0.03–0.07)
Gastrointestinal disorders				
​	Nausea/decreased appetite	6	18/2,088	0.01 (0.00–0.01), fixed
Skin and subcutaneous tissue disorders				
​	Skin irritation	2	5/490	0.01 (0.00–0.02), fixed
	Alopecia	2	6/768	0.01 (0.00–0.01), fixed
Investigations				
​	Weight gain	2	4/351	0.01 (−0.01–0.02), fixed

Abbreviations: AE, adverse effects; 95% CI, 95% confidence intervals.

## 4 Discussion

To our knowledge, this systematic review and meta-analysis is the first to synthesize evidence from 35 real-world studies and prove the efficacy and safety of BRV in adults with seizures. The results confirmed that BRV was beneficial for most the types of epilepsy when used both as monotherapy and as an adjuvant treatment in adults, providing further accurate and updated evidence supporting the clinical use of BRV for epilepsy management in day-to day adult clinical practice.

In this study, the primary efficacy outcomes of BRV were the pooled 50% responder and seizure-free rates over the treatment period. To date, six phase-II and phase-III trials have assessed the efficacy of BRV using the flexible-dose scheme (Biton et al., 2014; French et al., 2010; Klein et al., 2015; Kwan et al., 2014; Ryvlin et al., 2014; Van Paesschen et al., 2013). Among them, four trials indicated that the 50% responder rate of the BRV (5, 20, 25, 50, 100, and 150 mg/d) group ranged from 27.3% to 36.0%. Another phase-II-b study showed that the 50% responders in the 5, 20, and 50 mg/d BRV group were 32%, 44.2%, and 55.8%, respectively. The pooled 50% responder rate in our meta-analysis was 36% after 3 months of BRV treatment, regardless of its dosage, which was similar to those in RCTs. Interestingly, the pooled 50% responder rates were still as high as 35% and 40% when patients were treated with BRV at 6 and 12 months, indicating that BRV may be effective for long-term usage in routine clinical practice. Achieving complete seizure control without unacceptable AEs is a crucial target of epilepsy treatment that improves patients’ quality of life. A notable finding was the high seizure-free rate observed in our study. We found that the pooled seizure-free rates were 21.0% and 19.0% after 3 and 6 months of BRV treatment, even the long-term seizure-free rate was as high as 21.0%, which was much higher than those in RCTs. A high retention rate was observed in this study, indicating that BRV may be effective in real-world settings. Altogether, these results proved that the efficacy of BRV was similar to or even better than that of phase-II and phase-III trials. However, direct comparison of results between our study and the previous phase-II and phase-III trials is limited by the major differences in design and heterogeneous patient populations, necessitating cautious interpretation of the discrepancies. There are several factors that could affect the efficacy of BRV. All patients in RCTs were given a fixed-dose regimen, which might be the main reason for the reduced efficacy of BRV at lower doses. The seizure-free rate was significantly higher in real-world studies, but this result needs to be further tested and confirmed.

This study identified several factors that may affect the efficacy of BRV, providing valuable insights for prescribers regarding its clinical use. First, the current work confirmed that BRV was associated with greater sustained seizure control as measured by 50% responder and seizure-free rates, when used as an early add-on treatment rather than as a late add-on treatment. Consistent with our findings, previous studies demonstrated that patients with different ASMs were more likely to exhibit a higher response and seizure-free rates (Hou et al., 2023; Xiao et al., 2018). Additionally, patients still had a 30.8% chance of being 50% responder and a 10.8% chance of seizure freedom when BRV was taken as a late add-on treatment, even if they had tried ≥10 ASMs before BRV therapy. Thus, we recommend that early use of BRV could be considered if patients fail the first ASM since it appears to be more effective. Second, the current work confirmed the efficacy of BRV in the context of real-world clinical practice regardless of whether patients had previously used LEV; however, higher seizure-free rates were observed in patients without prior use of LEV. Nonetheless, patients still had a 33.1% chance of 50% responders even if they had a history of LEV usage and discontinued it due to insufficient efficacy or AEs, with similar 50% responder rates to those without previous treatment with LEV. Furthermore, we explored the safety and tolerability of immediately switching from LEV to BRV. A remarkable reduction in seizure frequency and lower AEs were found among patients who switched from LEV to BRV. Overall, our results from real-world clinical practice confirmed that although previous LEV failure can affect the response to BRV, treatment with BRV could still be of benefit for these patients and had a low rate of AEs. These interesting findings indicated that although BRV acts with a similar mechanism of action to that of LEV, they may have different mechanisms of action at their common binding target. These results are consistent with the findings of previous studies (Roberti et al., 2024; Steinhoff et al., 2020), which found that treatment with BRV could still be more effective for patients with prior LEV failure. Third, BRV used as monotherapy showed numerically better effectiveness for focal epileptic seizures, although all patients received PER after conversion to monotherapy. To date, few studies have reported clinical experience with a small number of patients receiving BRV as monotherapy. In addition to this, the data on BRV used as primary monotherapy are limited. In the current study, the pooled seizure-free rate from real-world studies was 73.3% when PER was administered as monotherapy at 6 months. Our results are encouraging, suggesting that BRV may be a valuable treatment for seizures in patients who converted to monotherapy in a real-life setting. Fourth, BRV treatment still showed some benefits in patients with LDs and epilepsy. Patients with LDs are often excluded from RCTs, so there is a lack of information on the best approach in such patients. To the best of our knowledge, the current research is the first to synthesize evidence from real-world clinical studies. The 50% responder rate in patients without LDs appeared to be marginally better than in those with LDs: 29% in the LD− group, which is similar to 24% in the LD+ group. However, numerically higher seizure-free rates were observed in patients without LD. This could be because patients in the LD group took more ASMs with BRV and presented highly refractory epilepsy, which is difficult to treat. These findings are similar to those of smaller studies that included patients with epileptic encephalopathies or LDs (Andres et al., 2018; Green et al., 2022).

The pooled AEs and withdrawal rates in the treatment period were analyzed as outcomes for measuring the safety of BRV. The pooled AE rates at 3, 6, and 12 months were 39%, 29.0%, and 34.0%, respectively. The pooled withdrawal rates due to AEs were 7.0%, 11.1%, and 10.0%, respectively. Our study indicated that BRV treatment was well-tolerated by patients with epilepsy. Furthermore, we assessed the pooled incidence of 24 AEs that were reported in clinical practice. The most frequently occurring AEs (reported in more than 50% of studies) involved the nervous system and psychiatric disorders, such as dizziness, somnolence, headache, depression, aggression, irritability, and fatigue. Most of the AEs of BRV were mild to moderate and could be alleviated after adjustment. Consistent with our results, a previous meta-analysis involving five third-generation ASMs demonstrated that the use of BRV as an add-on treatment was associated with a lower rate of AEs (Green et al., 2022). In addition, adults with epilepsy had the highest probabilities of having the best tolerance to BRV treatment (Green et al., 2022). However, it should be noted that the current study identified rare AEs requiring pharmacovigilance consideration, such as suicidal attempts, mental slowing, tremors, skin irritation, and alopecia. These rare AEs need to be tested in further studies.

## 5 Conclusion

In summary, the results of this meta-analysis from real-world studies provided new insights into the efficacy and safety of BRV usage as an adjunctive treatment and monotherapy for the majority of types of epilepsy, including those with learning difficulties, in a real-world setting. This study also demonstrated that BRV should be considered in patients with drug-resistant epilepsy (DRE) regardless of whether they previously used LEV. However, there was high heterogeneity in several pooled estimates in this article. Some factors could potentially impact the efficacy and safety of BRV as monotherapy or adjunctive treatment for adults with seizures, such as the type of epilepsy and the number of concomitant AEDs. Further studies involving larger cohorts and pharmacovigilance are needed to more extensively evaluate the clinical utility and profile of BRV monotherapy across various patient subgroups.

## Data Availability

The original contributions presented in the study are included in the article/Supplementary Material, further inquiries can be directed to the corresponding authors.
