# Supplementary material for: An evidence-based meta-analysis on the use of brivaracetam in treating seizures in real-world clinical practice
Source: Front Pharmacol. 2026 Jan 21;16:1716128. doi: 10.3389/fphar.2025.1716128 (PMC12868196; doi:10.3389/fphar.2025.1716128)
Supplement: Supplementary file 1 [file Supplementaryfile1.docx]

**Supplementary Table 1.** Literature retrieval strategy of the electronic databases.

**PubMed search strategy**

| Search number | Query | Results |
| --- | --- | --- |
| #5 | (#1 OR #2) AND (#3 OR #4) | 415 |
| #4 | "epilepsy"[Title/Abstract] OR "acute epilepsy"[Title/Abstract] OR "Awakening Epilepsy"[Title/Abstract] OR "chronic epilepsy"[Title/Abstract] OR "Cryptogenic Epileps*"[Title/Abstract] OR "epilepsi*"[Title/Abstract] OR "epileptic"[Title/Abstract] OR "epileptic disorder"[Title/Abstract] OR "epileptic syndrome*"[Title/Abstract] OR "falling sickness"[Title/Abstract] OR "seizure disorder*"[Title/Abstract] OR "tardy epilepsy"[Title/Abstract] | 28,889 |
| #3 | "Epilepsy"[Mesh] | 291,966 |
| #2 | Brivaracetam"[Title/Abstract] OR "Briviact"[Title/Abstract] OR "brivlera"[Title/Abstract] OR "nubriveo"[Title/Abstract] OR "rikelta"[Title/Abstract] OR "UCB 34714"[Title/Abstract] OR "UCB34714"[Title/Abstract] | 560 |
| #1 | "brivaracetam" [Supplementary Concept] | 262 |

**Embase Search Strategy**

| No. | Query | Results |
| --- | --- | --- |
| #6 | Limited to human, age≥12 | 615 |
| #5 | (#1 OR #2) AND (#3 OR #4) | 1216 |
| #4 | 'epilepsy'/exp | 304,027 |
| #3 | 'epilepsy':ti,ab,kw OR 'acute epilepsy':ti,ab,kw OR 'cryptogenic epileps*':ti,ab,kw OR 'awakening epilepsy':ti,ab,kw OR 'chronic epilepsy':ti,ab,kw OR 'epilepsi*':ti,ab,kw OR 'epileptic':ti,ab,kw OR 'epileptic disorder':ti,ab,kw OR 'epileptic syndrome*':ti,ab,kw OR 'falling sickness':ti,ab,kw OR 'seizure disorder*':ti,ab,kw OR 'tardy epilepsy':ti,ab,kw | 17,274 |
| #2 | 'brivaracetam'/exp | 1,505 |
| #1 | 'brivaracetam':ti,ab,kw OR 'briviact':ti,ab,kw OR 'brivlera':ti,ab,kw OR 'nubriveo':ti,ab,kw OR 'rikelta':ti,ab,kw OR 'ucb 34714':ti,ab,kw OR 'ucb34714':ti,ab,kw | 817 |

**Cochrane Central Register of Controlled Trials (CENTRAL) Search Strategy**

| ID | Search | Results |
| --- | --- | --- |
| #1 | (‘Brivaracetam’ OR ‘Briviact’ OR ‘brivlera’ OR ‘nubriveo’ OR ‘UCB 34714’ OR ‘UCB34714’):ti,ab,kw | 168 |
| #2 | MeSH descriptor: [Epilepsy] explode all trees | 3466 |
| #3 | (‘epilepsy’ OR ‘acute epilepsy’ OR ‘Awakening Epilepsy’ OR ‘chronic epilepsy’ OR ‘Cryptogenic Epileps*’ OR ‘epilepsi*’ OR ‘epileptic’ OR ‘epileptic disorder’ OR ‘epileptic syndrome*’ OR ‘falling sickness’ OR ‘seizure disorder*’ OR ‘tardy epilepsy’):ti,ab,kw | 8703 |
| #4 | #1 AND (#2 OR #3) | 138 |
