# Supplementary figures and images for: An evidence-based meta-analysis on the use of brivaracetam in treating seizures in real-world clinical practice
Source: Front Pharmacol. 2026 Jan 21;16:1716128. doi: 10.3389/fphar.2025.1716128 (PMC12868196; doi:10.3389/fphar.2025.1716128)

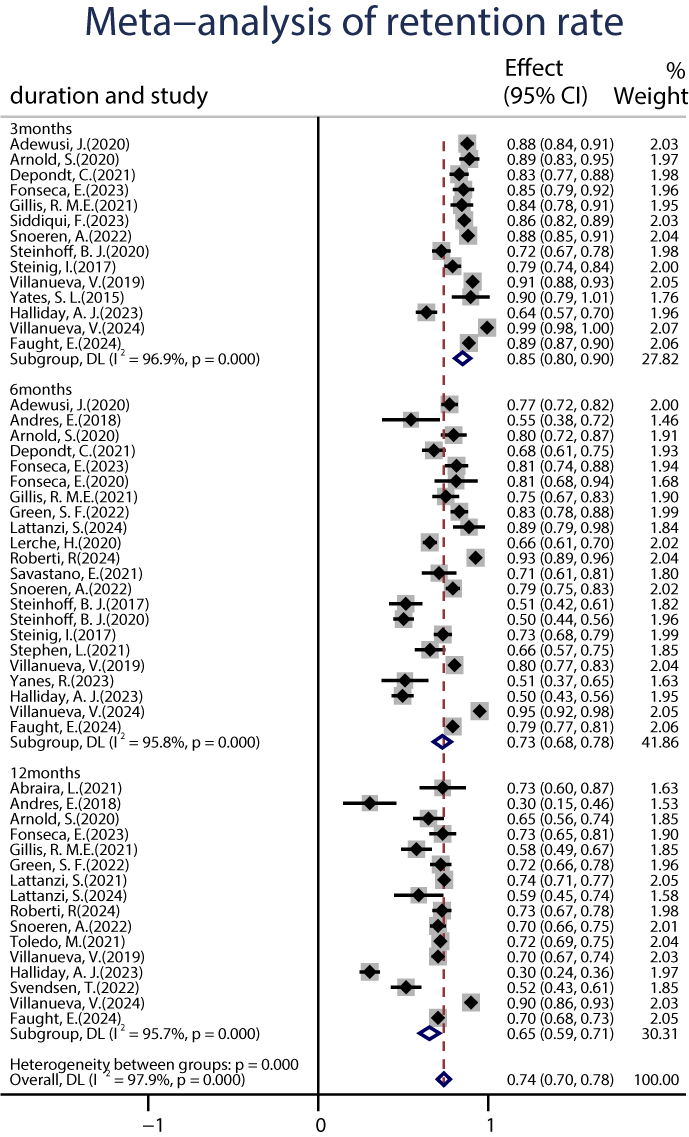

Supplement: Supplementary file 2 [file Image1.tif]
